# Objective Assessment of Attention Deficit Hyperactivity Disorder with QbMobile: A Smartphone Application for Clinical Use

**DOI:** 10.2174/0117450179444324251118134538

**Published:** 2025-11-22

**Authors:** Urban Gustafsson, Simon Larsson, Núria Casals, Robert Nolen, Ragini Yallampalli Sanyal, Mikkel Hansen

**Affiliations:** 1 Qbtech AB, Medical Department, Cardellgatan 1, 11436 Stockholm, Sweden; 2 Qbtech, Inc., Medical Department, 8 Greenway Plaza, Suite 750, Houston, TX 77046, United States

**Keywords:** Attention deficit hyperactivity disorder, Mental health interventions, QbMobile, QbTest, Psychometric test, Smartphone

## Abstract

**Introduction:**

Digital mental health interventions such as web or mobile applications have become more prominent in the last years to improve the clinical assessment and workflow in mental health disorders while also being potentially more accessible than laptops. QbMobile is a software application that provides objective assessments of hyperactivity, impulsivity, and inattention to aid in the clinical evaluation of attention deficit hyperactivity disorder (ADHD). The purpose was to examine whether QbMobile could objectively quantify symptoms and reveal significant clinical differences between an ADHD population and a normative population.

**Methods:**

Data were acquired from two low-intervention/observational studies (conducted in Europe and US), involving participants aged 6 to 60 years. The application (QbMobile) was configured on the smartphone (iPhone) with embedded instructions to ensure a consistent experience. Participants were seated at a desk in a stabilized chair and instructed to hold the smartphone with both hands and to tap the screen whenever an infrequent target stimulus appeared, while withholding a response to non-target stimuli. Concurrently, to measure activity, the camera of the smartphone captured the physical activity of the participant as well as the movements from holding the device. Approximately 20% of the complete dataset for each study was combined as a pooled dataset for a model validation of output parameters from QbMobile. A Total Score between 0 and 100 was calculated, where lower scores indicate a lower likelihood of ADHD symptoms and higher scores indicate a higher likelihood.

**Results:**

The ADHD cohort (n=63) demonstrated a higher mean Total Score of 83.0 (Standard deviation=17.5) compared to 48.9 (Standard deviation=18.8) in the normative population (n=354), a difference that was statistically significant (p<0.001). Significant domain-specific differences in SD-scores (movement pattern, activity, impulsivity, inattention) were identified between the ADHD cohort and the normative comparison group (p<0.001). A sensitivity of 0.86 and specificity of 0.75 were seen overall, though a low specificity was found in children, which was likely due to a smaller sample size and high activity levels in younger children in general.

**Discussion:**

This investigation demonstrates that QbMobile can generate objective symptom measurements that distinguish clinically relevant differences between individuals with ADHD and a normative population. A smartphone application of quantified behavioral psychometric testing of the core symptoms could streamline a faster diagnostic evaluation and treatment titration in the ADHD clinical workflow. The authors are employed by the manufacturer of QbMobile, which is discussed in this manuscript. This affiliation is disclosed to ensure transparency and does not affect the objectivity or scientific integrity of the work presented.

**Conclusion:**

QbMobile demonstrated the ability to differentiate between ADHD and normative cohorts, indicating its potential as an accessible and objective tool for clinical assessment and treatment evaluation. Future studies should be conducted to further validate the effectiveness of QbMobile as an aid tool in the clinical assessment of ADHD and further explore its use in various populations.

## 1 INTRODUCTION

Attention deficit hyperactivity disorder (ADHD) is a prevalent mental health condition affecting nearly 5% of children and 2.5% of adults globally [1-4], although these figures could be higher due to underdiagnosis [5]. The onset of ADHD occurs early, but a diagnosis is typically made at school-age [1, 3, 6]. The disorder ranks among the most common neurodevelopmental disorders with profound effects on personal, social, educational, and occupational functioning throughout the lifespan of that individual [7]. It is therefore crucial that individuals with probable ADHD have appropriate access to diagnosis as well as treatment.

ADHD can be addressed through a range of interventions such as targeted educational approaches and training programs [8, 9]. In recent years, mobile health applications have gained growing attention as innovative tools for supporting mental health and ADHD management [10, 11]. Using a smartphone for quantified behavioral psychometric testing is expected to be more accessible than, for example, laptops, as smartphones are more commonly available across a broader range of demographic and socioeconomic groups. While the designated hardware offers the same or better quality of captured data during the test, this increased accessibility supports more equitable participation in clinical assessment, research, and healthcare [12]. Mobile health apps on different platforms, such as iOS and Android, may thus provide attractive programs to help patients with access to ADHD information (for example, improving monitoring and rehabilitation) [13, 14], and may also enable tracking of treatment initiation, adherence, and effectiveness [15]. Mobile devices can provide healthcare professionals with rapid access to evidence-based information at the point of care and subsequently support better clinical decisions [11, 16]. Addressing the need to further improve assessment procedures, optimize treatment in ADHD, and create more options for equitable care, a smartphone-based test should therefore be of great interest in the clinical evaluation of ADHD [17]. Although in-clinic assessments continue to play an important role in standardized mental healthcare, mobile assessments offer an accessible alternative for individuals who face barriers to traditional care, such as geographic distance, limited local resources, or mobility constraints. At the same time, adapting to technology-based assessments may present challenges for some individuals; therefore, both in-person and mobile options remain important to ensure equitable access and engagement across diverse populations.

QbTest (in-clinic, office-based) and QbCheck (remote, home or clinic) are computerized assessments designed to objectively evaluate cognitive functioning and motor activity [18-23] of ADHD. These tests yield quantifiable data for the cardinal symptom domains of ADHD, such as inattention, hyperactivity, and impulsivity. QbTest and QbCheck are indicated for providing clinicians with objective measures of three cardinal symptom domains, thereby supporting the clinical assessment of ADHD and the evaluation of treatment effects [18, 20, 22, 24]. Developed as a possible solution to the challenges of equitable access, QbMobile is a smartphone-based software application that facilitates the objective test in a similar manner to QbTest and QbCheck. Installed on an iPhone, QbMobile captures participant responses via touchscreen taps and utilizes the smartphone’s camera to track movement-related activity during the testing procedure [25]. QbMobile test results were established in a general normative population, with the majority of participants reporting that QbMobile was user-friendly and achieved a strong degree of acceptance [25].

The aim of this investigation was to validate QbMobile, examine whether QbMobile could objectively quantify symptoms, and reveal significant clinical differences between ADHD and a normative population. This was done by using a pooled dataset for validation of the output from a trained machine learning model [26, 27] in a representative sample. The development of a mobile version of this assessment has the potential to streamline ADHD assessment and treatment workflows by enabling efficient, standardized data collection outside traditional clinic settings. Such an approach could not only enhance clinical decision-making but also extend access to mental health and wellness services for individuals in communities where in-person care is limited, ultimately supporting more equitable and scalable models of care.

## 2 MATERIALS AND METHODS

### 2.1 Data Collection and Participants

Data for the validation of the model were acquired from two low-intervention/observational studies QB21-01 (performed in the following locations: the United States, the Netherlands, and Germany) between April 2022 to August 2024, and QB22-01 (performed in the following locations: Germany, the Netherlands, the United Kingdom and the United States) between March 2022 to September 2023. Participants between 6-60 years were included in both studies. Participants for the clinical QB21-01 study were recruited from FocusMD, Alabama, USA; Phillipps-Universität, Marburg, Germany; and ADHDCentraal, Amsterdam, the Netherlands. For the QB21-01 study, the inclusion criteria were as follows: referred for initial ADHD assessment, no prior ADHD diagnosis, physically/sensorily able to complete QbMobile, and no psychostimulant use in the past month. The exclusion criteria were as follows: IQ<75, major medical/neurological conditions (*e.g.*, Parkinson’s, Alzheimer's disease), severe pain (*e.g.*, migraines), or substance use that could affect test performance. Determining a diagnosis of ADHD was made according to each participating site’s clinical standard assessment procedure, including measures such as ADHD symptom rating scales and DSM/ICD-based clinical interviews. For the normative QB22-01 study, a Clinical Research Organization was responsible for the recruitment of participants in 37 sites distributed in the US, the UK, Germany, and the Netherlands [25]. The following inclusion criteria were: No current/lifetime of ADHD or untreated ADHD, physically/sensorily able to complete QbMobile. The criteria for exclusion included: ADHD treatment in the past 30 days, medical/neurological conditions affecting test validity (*e.g.*, brain injury, uncontrolled psychiatric disorder), and substance use on the day of testing.

The studies QB21-01 and QB22-01 were carried out in compliance with the ethical standards in the Declaration of Helsinki, and the guidelines of Good Clinical Practice (Clinical investigation of medical devices for human subjects) (ISO 14155:2020), ICH-GCP, and any national or local regulations, as appropriate. For the QB21-01 study, Ethical approval was granted from Advarra IRB (Institutional Review Board), Colombia, Maryland, US (Pro00054910/QB21-01); Ethikkommssion Phillipps Universität, Marburg, Germany (2021-89k); and Medisch Ethische Toetsingscommissie AMC, Amsterdam, the Netherlands (NL81608.000.22/2022.0508). For the QB22-01 study, Ethical approval was obtained by UserWise IRB, The Alameda, San Jose, California, United States (no. QB22-01_08-11-2022). Before taking part in the study, all participants gave written informed consent.

### 2.2 QbMobile Test

The QbMobile test has been described elsewhere [25]. For both QB21-01 and QB22-01 studies: following installation on the iPhone, QbMobile application presented an instructional video and a brief practice test that had to be completed prior to starting the main assessment. During the test, every tap on the smartphone screen was registered as a response [25]. To measure activity, the camera of the smartphone captured participants' physical activity as well as movement from holding the device. The design of QbMobile combines a motion tracking system with high resolution, linked with a computerized Go/No-Go concept for children. This type of test is based on the concept of requiring participants to respond each time a circle appears on the screen while also refraining from responding when a crossed-out circle is presented [18-21, 24]. A one-back task paradigm is utilized for adolescents and adults, which involves four possible options of stimuli in which the target is defined as a stimulus that is exactly identical to the preceding stimulus in both color and shape [18-21, 24]. The stimuli were shown for 200 milliseconds in a two-second interval for 10 minutes. To support data collection, iPhones were distributed to all participating sites. The QbMobile application evaluated in this study was designed specifically for the iPhone in its initial development phase, with future iterations intended to extend compatibility across additional mobile platforms.

Apple’s ARKit [28] was employed for real-time tracking of the participant’s facial position in 3 dimensions during the execution of the objective test. The resulting time series data was subsequently processed to extract a series of features that captured the participant’s hyperactivity and movement patterns throughout the test duration.

The smartphone’s integrated sensors were utilized to monitor the participants’ movements while they held the device during the test. The accelerometer captured linear acceleration across three axes (x, y, and z), and the gyroscope measured rotational motion in terms of pitch, roll, and yaw. The time series data collected from each test were processed to generate a set of features aimed at capturing the activity and movement patterns observed during the test.

The QbTest software client was used to manage and analyze data from QbMobile, after which the data were transferred to a central server hosted by Amazon Web Services (AWS, Ohio). Transfers were encrypted, and analyses were restricted to pseudonymized data [25].

### 2.3 QbMobile Measurement

The data gathered from QbMobile is utilized to produce test results derived from several core parameters, which were established in the QbTest [18-21]. For activity, the factors include distance (the extent of facial movements throughout the test), determined in different time windows. Impulsivity utilizes measures of commission errors (occurs if a response is incorrectly made to a non-target stimulus), and error rate (frequency of incorrect responses) [18-21]. Inattention is evaluated through trends of both omission errors (occur when no response is made to a target stimulus) and reaction time (the delay before responding to a stimulus once presented) [18-21]. QbMobile also includes a movement pattern domain, which is based on face movements as well as movement of the smartphone itself (such as frequencies and timings) [18-21]. These variables are converted into measurable parameters and scores that form the analytic basis of the test results. Furthermore, QbMobile provides several parameters extracted from the motion capture camera, sensors, and the objective test. Some parameters are more related to each other in the sense that they measure the same underlying construct, which relates to the overall level of activity. The grouping of related parameters varies according to their correlation (factor loading) with the domain.

The data from QbMobile is presented as an SD-score for the domain. The SD-score is a standardized metric used to compare an individual's performance to a normative group. By definition, an SD-score of 1 indicates a deviation of one standard deviation from the normative reference group. Initially, when validating the normalization of SD-scores for the Norm group, the SD-scores were aimed to be set to zero. However, during the course of the revalidation and recalculation process of the model, the number of subjects (n) was increased to some degree, which had a very small impact on the Norm data (normalizations) SD-scores.

The Total Score is a calculated score from QbMobile. Total Score is used as a comparison between a group of normally developing individuals and individuals with a clinical diagnosis of ADHD and has been validated by an external validation data set. It is expressed as the likelihood for the test-taker to belong to the clinical group. This variable can be used in the assessment of a test taker evaluated for ADHD, providing the best possible classification based on all the data the model has access to. The Total Score is a number between 0 and 100 that reflects the model’s evaluation of how likely an individual is to exhibit ADHD symptoms, with 0 indicating low likelihood and 100 indicating high likelihood. The Total Score and Domain scores are the primary measures used to interpret the QbMobile results. Activity, Impulsivity, Inattention, and Movement Pattern each consider various underlying variables from the QbMobile model, making them more robust than any individual underlying measure.

### 2.4 Data and Statistical Analysis

QB21-01 study was conducted to collect data on QbMobile client usability and data acquisition ability and comparability to QbTest. The QB22-01 study was conducted to establish a normative cohort database for QbMobile. The two resulting cohorts were divided into two subsets; approximately 80% of the data is kept for model training, while the residual 20% is kept for the test set to facilitate a fair assessment of the model. The 80/20 division was performed using randomized stratification to ensure that the process preserved the class distribution (ADHD vs. normative). Data splitting allows for robust training of models to identify patterns in ADHD data while ensuring that the model can accurately predict outcomes in new, unseen cases [29, 30]. The validation cohort (20%) was the basis for confirmation of the output parameters presented in this investigation.

The Total Score is a calculated metric of how likely a patient is to have ADHD-like symptoms (where 0 is low and 100 is high likelihood of having ADHD symptoms). To compute such a score, distinct types of features were created to reflect the most common symptoms of ADHD. These features were fed into a set of machine learning algorithms that use mathematical optimization to find the most informative patterns for classifying individuals with and without ADHD [26]. Sensitivity and specificity were the chosen metrics to test the classification ability of the Total Score. These metrics capture the model’s ability to correctly classify the positive labels (ADHD) and the negative (Norm). Choosing sensitivity and specificity as metrics is motivated by the broad acceptance and frequent use of these measures in clinical studies where positive and negative labels are not balanced. The model was considered successfully validated when sensitivity and specificity reached levels comparable to or above the commonly used threshold of 0.8.

Machine learning employs various statistical, probabilistic, and optimization techniques to discover meaningful patterns in large, complex, and unstructured data [27]. The model falls into the binary classification category. The model learns from labelled data that contains test information and the diagnosis of two different cohorts. The goal was to create a mapping from input features to output labels (ADHD or Norm) so that when presented with new, unseen data, the algorithm could accurately predict the corresponding output labels with a Total Score.

A wide range of machine-learning methods is available, but the present analysis focused on models offering a reasonable degree of interpretability. Tree-based approaches, which partition data into progressively smaller subsets based on predictor values, were of particular interest. Algorithms in this family, such as decision trees, random forests, and gradient-boosting methods, provide flexible predictive frameworks while retaining some transparency [31, 32]. Group mean differences were evaluated using an independent-samples t-test, a well-established statistical procedure suitable even when sample sizes differ. Model selection was guided by cross-validation performance and the ability to handle complex, non-linear relationships in the data. To account for the imbalance between ADHD and normative participants, Precision-Recall Area Under the Curve (PR-AUC) was used as the primary training evaluation metric, as it is especially informative for class-imbalanced predictive tasks due to its sensitivity to changes in false positive rates [33]. Stratified K-fold cross-validation was used to ensure that each split maintained the original class distribution, reducing potential bias due to class imbalance in the model evaluation. Classification thresholds were chosen to maximize balanced accuracy across the sample.

The sample size was based on N=2338 in order to have a satisfactory cohort for both model training and validation. The reported model validation cohorts (n=417; of which norm n=354 and ADHD n=63, for which data are presented in this publication) represent an approximation of 20% of the original dataset. Descriptive statistics were calculated and presented with count (N), percentage (%), mean and standard deviation, and min-max, as appropriate for categorial and continuous variables. Statistical significance was assessed at the conventional threshold of p < 0.05. Comparisons of group means (ADHD vs. normative) were performed using a two-tailed Welch’s t-test, which accounts for unequal variances [34]. Cohen’s d was used to assess effect size between groups and was assessed using a widely used guideline of d = 0.2 for a small effect, d = 0.5 for a medium effect, and d = 0.8 for a large effect [35]. Sensitivity and specificity were analyzed using standard formulas.

## 3 RESULTS

### 3.1 Demographics

The model validation consisted of a total of N=417 participants. The data for the normative cohort was based on N=354 participants in the age group covering 6 to 60 years (overall), of which 30 participants were between 6 to 11 years (children), and 324 participants were in the age group 12-60 years (adolescents/adults). The data for the ADHD cohort was based on N=63 participants within the studied 6-to-60-year age group (overall), of which 15 participants belonged to the 6-to-11-year age group (children) and 48 participants were in the age group 12-60 years (adolescents/adults). Overall, there were more females than males in the normative cohort (58.8% vs 41.2%) as well as in the ADHD cohort (57.1% vs 42.8%). Demographics (sex, race, and ethnicity) were generally considered similar between the normative and ADHD cohorts, with the majority of participants identifying as White regarding ethnicity (Table **1**). The normative and ADHD cohorts showed comparable characteristics in terms of vision and eye color (Table **1**). Descriptive analyses of measures derived from ARKit-based smartphone face tracking showed no impact by race, vision, or eye color [25].

The dataset analyzed was diversely represented with participants from the United States and three European countries (Germany, the United Kingdom, and the Netherlands) (Table **2**).

### 3.2 Total Score

Mean and standard deviation of Total Score (0-100), as well as the Total Score distributions versus the number (N) of participants, for the normative (Norm) cohort and ADHD cohort are given in Table **3** and Fig. (**1**). The ADHD cohort (n=63) demonstrated a higher mean Total Score of 83.0 (Standard deviation=17.5) compared to a mean of 48.9 (Standard deviation=18.8) in the normative population (n=354), a difference that was statistically significant (p<0.001) and reflected a very large effect size (Cohen’s d = 1.83).

The ADHD cohort demonstrated a higher mean Total Score, also when split by age or sex groups. The differences in distribution were statistically significant (p<0.001), and the effect sizes were large (Cohen’s d from 1.64 to 1.95) for all splits.

### 3.3 Domain Scores

All feature groups - movement pattern, activity, impulsivity, and inattention - contributed significantly to the machine learning model. Significant domain-specific differences in SD-scores were identified between the ADHD cohort and the normative comparison group (p<0.001) (Table **4**). Effect sizes (Cohen’s d) varied from 0.53 to 1.63, indicating medium to very large differences between the ADHD and normative groups across all domains.

An example of a presentation of a domain SD-score from a QbMobile test in comparison to the normative group and the ADHD group is shown in Fig. (**2**). When a test taker is performing a QbMobile test, age and sex are taken into consideration and impact the SD-score presentation of the respective domain in comparison to the normative distribution.

### 3.4 Sensitivity and Specificity

A sensitivity of 0.86 and specificity of 0.75 were seen overall (Total 6-60 years), and by age group and sex are also given in Table **5**. Notably a low specificity of 0.43 was found in the Child group (6-11 years), which was limited by the small sample size (n=15) and high activity levels in younger children in general.

### 3.5 Safety

No adverse events or any safety concerns were reported during the administration of the QbMobile device.

## 4 DISCUSSION

The findings from this investigation concluded that QbMobile, a smartphone application of QbTest, was found to be able to distinguish between ADHD and normative cohorts. A significant difference in Total Score as well as in the four domains of QbMobile (movement pattern, activity, impulsivity, and inattention) was demonstrated between individuals with ADHD and the normative population. With the ability to identify clinically relevant differences between ADHD and normative cohorts, QbMobile is positioned to be an accessible and objective tool for clinical assessment and treatment evaluation.

To assess how well a diagnostic test performs, both sensitivity and specificity need to be taken into account [36, 37], for which sensitivity is the probability that the test correctly classifies individuals with the disorder, while specificity relates to the test's ability to correctly identify participants without the disorder [36, 37]. The Total Score achieved a sensitivity of 0.86 and specificity of 0.75 with a combined score of 0.8, which aligns closely with the predetermined and expected goal of 0.8. These sensitivity and specificity values indicate a good and reasonable diagnostic accuracy, respectively [36, 37]. The found sensitivity and specificity are also in line with similar devices, QbTest and QbCheck, with a sensitivity and specificity of 0.89 and 0.87, and 0.83 and 0.80, respectively [20, 23]. However, a low specificity was seen in the Child group (0.43), which was likely due to a smaller sample size, and a low specificity can render a higher proportion of false positive results. Moreover, the main diagnostic outputs from QbMobile are the classification score (Total Score) and the SD-scores of the four domains (activity, impulsivity, inattention, and movement pattern), all of which had a very large to medium effect size and statistical p-values well below 0.01, meaning there was a significant difference between the means. Hence, the descriptive power of the Total Score and domain SD-scores was found to be positively validated, which is considered clinically meaningful.

Digital health tools such as mobile applications have vastly improved access to healthcare services and information [38]. These tools empower both healthcare providers and patients by offering real-time data that can facilitate more efficient diagnosis and treatment management [39]. Mobile applications also enable patients to monitor their symptoms and identify appropriate health care specialists, potentially reducing unnecessary clinic visits and optimizing healthcare resources [11]. However, barriers such as stigma, limited provider capacity, prolonged wait times, and geographical restrictions can impede individuals with mental health conditions from accessing or pursuing appropriate care [40]. Mental health mobile apps may help bridge this gap by expanding access to mental health and wellness services for underserved populations [41]. Digital interventions delivered via smartphones, such as organizational skills training, may help subjects with ADHD develop more structure in their daily lives, while telemedicine platforms offer new opportunities to deliver clinical care and improve outcomes for this population [42]. Furthermore, mobile mental health apps may represent a cost-effective approach for patients with ADHD and their caretakers [43], although additional real-world studies are warranted to confirm their cost-effectiveness in clinical practice [44].

Several overviews have shown promising capabilities of CPTs and objective measures to distinguish between ADHD and healthy controls, as well as provide useful information on a person’s behavior, which can potentially enhance diagnostic decisions and decrease assessment times in the clinical workflow [45-50]. Hall *et al.* (2016) stated that robust support for objective activity measures in distinguishing ADHD from non-ADHD populations exists, with evident sensitivity to medication effects, which, if evaluated, could show potential for additional clinical utility [51]. Objective measures may therefore provide a robust framework for assessing ADHD symptoms, offering greater precision and consistency compared to traditional methods. In this context, QbMobile should have a place as a versatile tool and a clinically accessible objective measure useful for ADHD assessment and the evaluation of treatment efficacy.

Objective measurements of the cardinal symptoms are essential for proceeding with the diagnosis and clinical management of ADHD. Hyperactivity is characterized by excessive motor activity when it is not appropriate or excessive fidgeting. Inattention refers to difficulties in sustaining focus or completing tasks. Impulsivity implies acting without forethought and difficulty inhibiting inappropriate answers [52]. QbMobile has an added domain including Movement patterns in this perspective, which is based on face movements and motions of the smartphone. It is widely recognized that children with ADHD experience multiple motor skill deficits, such as poorer handwriting, reduced coordination and motor control, difficulty in motor planning, and less accurate, often jerky or uncoordinated movements [53]. The rate and sequencing of movements also appear to be altered. Though fine motor deficits appear to be independent of sex [54], this cannot completely be ruled out [55]. No effect on handedness appears to be present, as motor control impairments are evident in both the dominant and non-dominant hands [56]. Impairments are also evident in both gross and fine motor skills [54]. Although ADHD itself does not cause involuntary movements, available evidence indicates that unintentional movements, for example, tics and other motor abnormalities, are more common in individuals with ADHD compared to the general population [57].

The current version of QbMobile uses a static machine learning model. It is a deliberate design choice that aligns with clinical and regulatory standards. A static model ensures reproducibility, interpretability, and consistent calibration, while minimizing risks associated with unverified model updates. This stability provides greater control and reliability in medical settings, where patient safety and model transparency are essential. Another key advantage is the potential for future updates as more data becomes available. While traditional scoring systems can also be revised, machine learning models are better suited to improve performance as they scale with larger and more diverse datasets [58, 59]. This enables more precise identification of ADHD-related behavioral and cognitive patterns across different populations [60, 61]. Machine learning also offers the flexibility to incorporate additional data sources, including expanded sensor inputs, contextual information about the individual, and longitudinal patterns captured by the smartphone outside the immediate test window, all of which may further enhance diagnostic accuracy [62, 63]. Additionally, periodic model updates can help maintain clinical validity by accounting for shifts in user behavior, technology use, or population characteristics over time. While continuous learning is not currently implemented, the digital format of QbMobile allows for retraining and deployment of updated models. However, any improvements must be accompanied by rigorous validation to ensure clinical reliability and compliance with regulatory and diagnostic standards [64].

### 4.1 Limitations

The authors are employed by the manufacturer of QbMobile, which is discussed in this manuscript. This affiliation is disclosed to ensure transparency and does not influence the objectivity or scientific integrity of the work presented.

The sample size for the validation in this investigation was considered satisfactory overall. However, a small sample size was seen in the younger ADHD age group (children), which could hamper the data in comparison to the normative cohorts. This should be considered and may limit the generalizability of the findings to the broader population, as it may introduce bias, including selection bias, overfitting, and limited generalizability [65]. The sample size limitation underscores the need for replication in larger and independent cohorts. Furthermore, as part of a continuous improvement model, future studies should further validate the effectiveness of QbMobile as an aid tool in the clinical assessment of ADHD, which will support the expansion of the dataset over time, ensuring that findings continue to reflect a growing and increasingly representative sample.

A limitation of the current model is the imbalanced sensitivity and specificity observed in children, despite a large effect size indicating clear group-level separation between ADHD and normative participants. This pattern suggests that while the model effectively captures meaningful differences in underlying signal characteristics, the classification threshold may not be well calibrated for younger individuals. In other words, the model tends to over-identify ADHD cases in children, likely due to developmental or behavioral variability that shifts score distributions relative to adults. Classification thresholds were chosen to maximize balanced accuracy across the full sample regardless of age range. While this approach ensures overall comparability, it may contribute to reduced specificity in pediatric participants. Future work should explore age-specific calibration or adaptive thresholds to improve diagnostic precision in children. Incorporating developmental or behavioral factors might help account for variability in model scores across ages. Longitudinal studies could further clarify how predictions change as children grow, allowing thresholds to be adjusted over time. Finally, expanding the pediatric sample and validating the model in independent child cohorts would help ensure the findings are robust and generalizable.

## CONCLUSION

The aim was to investigate whether QbMobile, a smartphone application of QbTest, is able to generate objective symptom metrics that demonstrate significant differentiation between individuals with ADHD from a normative population in a model validation. A statistical difference in Total Score was found between the ADHD and the normative cohorts as well as across the four QbMobile domains (movement pattern, impulsivity, inattention, and activity). The model reached a sensitivity of 0.86 and specificity of 0.75. These results show that QbMobile can be used to improve the accuracy and objective identification of ADHD symptoms to facilitate diagnostic assessment and treatment interventions for ADHD. A mobile application of quantified behavioral psychometric testing of ADHD core symptoms could accelerate diagnostic evaluation, improve treatment titration, streamline clinical workflows, and help bridge the gaps in access to quality mental health services. Future studies should be conducted to further validate the effectiveness of QbMobile as an aid tool in the clinical assessment of ADHD and further explore its use in various populations.

## Figures and Tables

**Fig. (1):**
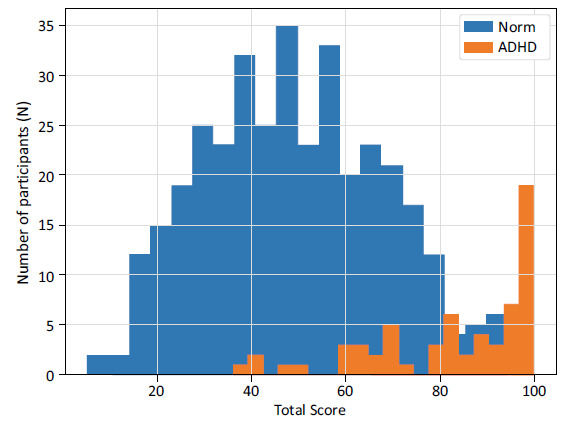
Total Score (from QbMobile) (range 0-100, where 0 indicating low and 100 indicating high likelihood of having ADHD symptoms) distribution (x-axis) versus the number (N) of test takers (y-axis) for the normative (Norm) cohort (blue) (n=354) and the ADHD cohort (orange) (n=63).

**Fig. (2):**
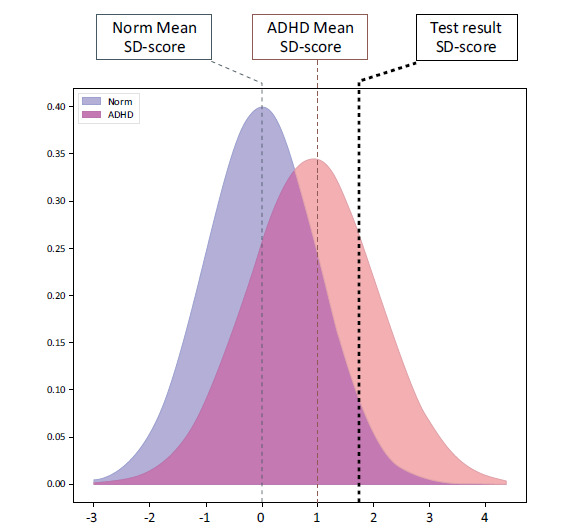
An example of a QbMobile domain SD-score illustration, presented as the probability density function of the normative (Norm) (blue) and ADHD (red) distribution. On x-axis is the SD-score with Standard deviation scaling for which the normative distribution is set to zero, and on y-axis is the density of the corresponding distribution. The vertical dotted line (black) shows an example of an SD-score outcome from a test taker with ADHD.

**Table 1:** Demographics and characteristics for the normative (Norm) cohort and the ADHD cohort for the different age groups (6-11, 12-60 and 6-60 years) (number of participants).

*Demographics*	Norm Age group 6 to 11 years (n=30)	ADHD Age group 6 to 11 years (n=15)	Norm Age group 12 to 60 years (n=324)	ADHD Age group 12 to 60 years (n=48)	Norm (Total) Age group 6 to 60 years (n=354)	ADHD (Total) Age group 6 to 60 years (n=63)
**Age (Years)**						
Mean	9.2	9.7	33.3	27.4	31.3	23.2
Standard deviation	1.7	1.3	13.0	10.0	14.2	11.6
Min	6	7	12	13	6	7
Max	11	11	58	52	58	52
**Sex**						
Male	10 (33.3%)	9 (60.0%)	136 (42.0%)	18 (37.5%)	146 (41.2%)	27 (42.8%)
Female	20 (66.7%)	6 (40.0%)	188 (58.0%)	30 (62.5%)	208 (58.8%)	36 (57.1%)
**Race**						
White	13 (43.3%)	9 (60.6%)	196 (60.0%)	39 (81.2%)	209 (59.0%)	48 (76.2%)
Black	9 (30.0%)	3 (20.0%)	50 (15.4%)	1 (2.1%)	59 (16.7%)	4 (6.3%)
Asian	1 (3.3%)	0	41 (12.7%)	0	42 (11.9%)	0
Other	7 (23.4%)	3 (20.0%)	37 (11.4%)	8 (16.7%)	44 (12.4%)	11 (17.5%)
**Ethnicity**						
Not Hispanic Latina/Latino, or Spanish descent	24 (80.0%)	14 (93.3%)	258 (79.6%)	39 (81.2%)	282 (79.7%)	53 (84.1%)
Hispanic, or Latino/a, or Spanish origin	4 (13.3%)	1 (6.7%)	19 (5.9%)	2 (4.2%)	23 (6.5%)	2 (3.2%)
Prefer not to say	0	0	9 (2.8%)	1 (2.1%)	9 (2.5%)	1 (1.6%)
Unknown	2 (6.7%)	0	38 (11.7%)	6 (12.5%)	40 (11.3%)	7 (11.1%)
***Characteristics***						
**Vision**						
Normal	25 (83.3%)	12 (80.0%)	200 (61.7%)	22 (45.8%)	225 (63.6%)	34 (54.0%)
Glasses	5 (16.7%)	3 (20.0%)	85 (26.3%)	17 (35.4%)	90 (25.4%)	20 (31.7%)
Contact Lenses	0	0	35 (10.8%)	7 (14.6%)	35 (9.9%)	7 (11.1%)
Other	0	0	4 (1.2%)	2 (4.2%)	4 (1.1%)	2 (3.2%)
**Eye Color**						
Brown	20 (66.6%)	6 (40.0%)	172 (53.1%)	17 (35.4%)	192 (54.2%)	23 (36.5%)
Blue	6 (20.0%)	5 (33.3%)	82 (25.3%)	20 (41.7%)	88 (24.9%)	25 (39.7%)
Green	2 (6.7%)	1 (6.7%)	46 (14.2%)	7 (14.6%)	48 (13.6%)	8 (12.7%)
Hazel	2 (6.7%)	1 (6.7%)	7 (2.2%)	0	9 (2.5%)	1 (1.6%)
Black	0	0	3 (0.9%)	0	3 (0.9%)	0
Other	0	2 (13.3%)	14 (4.3%)	4 (8.3%)	14 (3.9%)	6 (9.5%)

**Table 2:** Participants by country for the normative (Norm) cohort and the ADHD cohorts (number of participants).

**Country**	**Norm (n=354)**	**ADHD (n=63)**	**Total (n=417)**
Germany	59 (16.7%)	14 (22.2%)	73 (17.5%)
The Netherlands	62 (17.5%)	16 (25.4%)	78 (18.7%)
United Kingdom	137 (38.7%)	0	137 (32.9%)
United States	96 (27.1%)	33 (52.4%)	129 (30.9%)
Total	354 (100%)	63 (100%)	417 (100%)

**Table 3:** **QbMobile** Total Score (Mean and Standard deviation) and effect size by age group and gender for the Normative (Norm) cohort and the ADHD cohort (n= Number of participants).

Age group/gender	Norm (N)	ADHD (N)	Norm Mean	Norm Standard deviation	ADHD Mean	ADHD Standard deviation	T-test p-value	Effect size (Cohen’s d)
All (age 6-60 years)	354	63	48.9	18.8	83.0	17.5	<0.001	1.83
Adult (12-60 years)	324	48	47.4	18.3	80.8	18.5	<0.001	1.82
Child (6-11 years)	30	15	64.9	16.7	90.1	11.8	<0.001	1.64
Male	146	27	47.9	17.9	82.8	17.4	<0.001	1.95
Female	208	36	49.6	19.4	83.2	17.8	<0.001	1.72

**Table 4:** The QbMobile SD-scores by domain for the normative (Norm) cohort and the ADHD cohort (n=number of participants). Values are presented as Mean or Standard deviation in respectively column together with statistical significance and effect size.

**Domain**	**Norm** **Mean** **(SD-score) (n=354)**	**Norm Standard deviation** **(SD-score)**	**ADHD** **Mean** **(SD-score)** **(n=63)**	**ADHD** **Standard deviation** **(SD-score)**	**T-test** **p-value**	**Effect size** **(Cohen’s d)**
**Activity**	-0.03	0.93	1.13	1.23	<0.001	1.18
**Impulsivity**	-0.16	1.05	0.44	1.10	<0.001	0.57
**Inattention**	-0.24	0.24	0.87	1.68	<0.001	1.63
**Movement pattern**	0.27	1.17	0.89	1.21	<0.001	0.53

**Table 5:** Sensitivity and specificity of QbMobile by age groups, gender and overall (6-60 years) for the normative (Norm) (n=354) and the ADHD (n=63) cohorts.

**Age group/gender**	**Norm (N)**	**ADHD (N)**	**Sensitivity**	**Specificity**
Adult (12-60 years)	324	48	0.83	0.78
Child (6-11 years)	30	15	0.93	0.43
Male	146	27	0.81	0.79
Female	208	36	0.89	0.72
Overall (6-60 years)	354	63	0.86	0.75

## Data Availability

The authors confirm that the data supporting the findings are available within the article. Due to the nature of the research, limited access to supporting data may be granted upon reasonable request under confidentiality agreements from the corresponding author.

## LIST OF ABBREVIATIONS

ADHD: Attention Deficit Hyperactivity Disorder
SD-score: A SD-score of 1 is equivalent to 1 standard deviation from the normative cohort

## STANDARDS OF REPORTING

STROBE guidelines were followed.
